# Immunomodulatory effects of regorafenib: Enhancing the efficacy of anti-PD-1/PD-L1 therapy

**DOI:** 10.3389/fimmu.2022.992611

**Published:** 2022-09-02

**Authors:** Junjie Liu, Haisu Tao, Tong Yuan, Jiang Li, Jian Li, Huifang Liang, Zhiyong Huang, Erlei Zhang

**Affiliations:** ^1^ Hepatic Surgery Center, Tongji Hospital, Tongji Medical College, Huazhong University of Science and Technology, Wuhan, China; ^2^ Hubei Key Laboratory of Hepato-Pancreato-Biliary Diseases, Tongji Hospital, Tongji Medical College, Huazhong University of Science and Technology, Wuhan, China; ^3^ Department of Hepatobiliary Surgery, Zhujiang Hospital, Southern Medical University, Guangzhou, China

**Keywords:** regorafenib, PD-1/PD-L1, immunotherapy, resistance, combination therapy, tumor microenvironment, immunomodulatory effects

## Abstract

Anti-PD-1/PD-L1 therapy has shown significant benefits in the treatment of a variety of malignancies. However, not all cancer patients can benefit from this strategy due to drug resistance. Therefore, there is an urgent need for methods that can effectively improve the efficacy of anti-PD-1/PD-L1 therapy. Combining anti-PD-1/PD-L1 therapy with regorafenib has been demonstrated as an effective method to enhance its therapeutic effect in several clinical studies. In this review, we describe common mechanisms of resistance to anti-PD-1/PD-L1 therapy, including lack of tumor immunogenicity, T cell dysfunction, and abnormal expression of PD-L1. Then, we illustrate the role of regorafenib in modifying the tumor microenvironment (TME) from multiple aspects, which is different from other tyrosine kinase inhibitors. Regorafenib not only has immunomodulatory effects on various immune cells, but can also regulate PD-L1 and MHC-I on tumor cells and promote normalization of abnormal blood vessels. Therefore, studies on the synergetic mechanism of the combination therapy may usher in a new era for cancer treatment and help us identify the most appropriate individuals for more precise treatment.

## 1 Introduction

In recent years, immune checkpoint inhibitors (ICIs) have achieved considerable progress in the immunotherapy of cancer. ICIs act on a suppressed immune system and activate CD8^+^ T cells to attack tumor cells (1). ICIs are now being researched in clinical studies for a range of malignancies (2, 3). However, there are still some patients who cannot benefit from anti-PD-1/PD-L1 therapy due to the development of resistance in the tumor (4). In past studies, researchers have focused mostly on how immune cells combat tumor cells face-to-face. Nevertheless, it is also essential to consider whether the environment in which this fight takes place is conducive to immune cell activity. Combining anti-PD-1/PD-L1 therapy with other therapeutic medications that help improve the TME may be an effective method to enhance its therapeutic potential.

Regorafenib is a tyrosine kinase inhibitor that is used in patients with colorectal cancer (CRC) (5), gastrointestinal stromal tumor (GIST) (6), and hepatocellular carcinoma (HCC) (7). Regorafenib differs from sorafenib in that a fluorine atom has been added to the central phenyl ring, making it more pharmacologically active than sorafenib (8). Recent studies have shown that regorafenib can optimize the efficacy of anti-PD-1/PD-L1 treatment. Regorafenib combined with nivolumab showed good efficacy and tolerable safety in the treatment of CRC (9). Therefore, regorafenib combined with ICIs therapy may be a promising approach for cancer therapy.

## 2 The underlying mechanism of resistance to PD-1/PD-L1 blockade

### 2.1 PD-1 and its ligands

PD-1 (also known as CD279) encodes a 50-55 kDa protein that is a member of the CD28/cytotoxic T-lymphocyte antigen 4 (CTLA4) family (10). PD-1 was originally isolated from the murine T-cell hybridoma cell line 2B4.11 and is thought to play a role in programmed cell death (11). However, subsequent studies revealed that the expression of PD-1 may not be necessary for apoptosis (12). PD-1 deficiency was found to be associated with autoimmune diseases in PD-1-deficient C57BL/6 mice (13). Furthermore, selective upregulation of PD-1 was also observed on exhausted T cells (14). PD-L1 (also known as CD274), which belongs to the B7 gene family (15), is expressed in many immune cells and non-hematopoietic cells, including macrophages, dendritic cells (DCs), B cells, T cells, vascular endothelial cells, hepatocytes, and epithelial cells (16). PD-1 binds to its ligand, PD-L1 or PD-L2, suppresses T-cell receptor (TCR)-mediated phosphorylation of ZAP70 and CD3zeta by recruiting protein tyrosine phosphatase SHP-2, inhibits downstream PI3K/AKT pathways, and transmits negative regulatory antigen receptor signals (17, 18). Similarly, the effect of suppressing B cell receptor (BCR) signaling was observed in B cells (19). In a variety of tumors, the overexpression of PD-1 on tumor-infiltrating immune cells or PD-L1 on tumor cells corresponds with a poor prognosis (20). The binding of PD-1 to PD-L1 is a potential mechanism through which tumors escape the host immune response, and eliminating this interaction can play a pivotal role in tumor immunotherapy (21, 22). Therefore, anti-PD-1/PD-L1 therapy has become an essential cancer therapeutic strategy. Currently, anti-PD-1/PD-L1 therapy has been approved for the treatment of various tumors, including CRC, HCC, non-small cell lung cancer (NSCLC), renal cell carcinoma (RCC), as well as head and neck cancer (HNC), among others (23).

### 2.2 Resistance to PD-1/PD-L1 blockade

Although anti-PD-1/PD-L1 therapy has great therapeutic prospects in a number of malignancies, there are still many patients who do not benefit from the treatment. In some cancers, primary treatment resistance is observed in more than 60% of patients (24). In non-responsive patients, PD-1/PD-L1 blockade alone is insufficient for cancer treatment. Before utilizing anti-PD-1/PD-L1 therapy, it is vital to evaluate which patients are most suited for this therapy and how to combine it with other treatment strategies. Therefore, it is crucial to understand why drug resistance occurs. Resistance to PD-1/PD-L1 blockade can be classified as primary or acquired treatment resistance (25). Patients who do not initially demonstrate a clinical response to anti-PD-1/PD-L1 therapy are considered to have “primary resistance” to therapy. For example, insufficient antigen immunogenicity and dysfunction of antigen presentation may affect the ability of APCs to present tumor rejection antigens to CD8^+^ T cells and thus affect the subsequent anti-tumor effects. “Acquired resistance” refers to the phenomenon in which initially immunotherapy-responsive tumor patients acquire treatment resistance or relapse over time. Due to the heterogeneity of tumors, tumor cells with low antigen expression and the upregulation of certain immunosuppressive signaling receptors may result in the development of acquired resistance (25, 26). Mechanistically, resistance to drugs targeting PD-1/PD-L1 can be due to the following aspects: 1) lack of tumor immunogenicity, 2) T cell dysfunction, 3) abnormal expression of PD-L1.

#### 2.2.1 Lack of tumor immunogenicity

Neoantigens produced by tumor cells can activate the immune response of T cells, which is related to stronger immunogenicity and is one of the targets of tumor immunotherapy (27). Firstly, the tumor mutational burden (TMB) can predict the effect of ICIs. A lower TMB tends to be correlated with worse clinical outcomes (28). Secondly, in some mismatch repair deficient cancers, neoantigen expression makes them more sensitive to ICIs (29). Conversely, mismatch repair proficient tumors are insensitive to ICIs. Thirdly, Β2-microglobulin (B2M) mutations lead to the downregulation of MCH-I, triggering the escape of tumor cells from treatment with ICIs (30, 31). All of the above are primary drug resistance. Fourthly, due to the existence of tumor heterogeneity, during the use of ICIs, tumor cells with low antigen expression are selected for continued growth, resulting in the emergence of acquired drug resistance (32).

#### 2.2.2 T cell dysfunction

Multifaceted T cell dysfunction may result in resistance to ICIs. Antigen-presenting cells (APCs) stimulate the activation of tumor antigen-specific effector T cells, whereas a lack of APCs or their inactivation inhibits effector T cell activation (33). In melanoma cells, activation of the WNT/Β-catenin pathway reduces the number of tumor-infiltrating lymphocytes (TILs) because activation of this pathway decreases the expression of chemokine C-C motif ligand 4 (CCL4), which is essential for tumor infiltration by DCs. Insufficient DCs for the activation of T cells results in resistance to ICIs therapy (34). Moreover, Foxp3^+^ regulatory T cells (Tregs) suppress the immune response to autoantigens to prevent the development of autoimmune diseases and also suppress the anti-tumor immune response (35). CTLA4 blockade can inhibit Tregs, reactivate the repressed T cells in the TME, and overcome the inhibitory immune microenvironment (36). As an immunosuppressive immature myeloid cell, myeloid-derived suppressor cells (MDSCs) also promote tumor invasion and contribute to tumor immune escape (37). The massive accumulation of MDSCs in tumor tissues will limit the inhibitory effect of ICIs on tumors (38). In some solid tumors, tumor-associated macrophages (TAMs) infiltration is strongly correlated with a poorer prognosis (39–41). TAMs play an essential role in strengthening angiogenesis, tumor growth and metastasis, as well as dampening anti-tumor immunity (42). These suppressive immune cells impair the normal function of T cells, leading to primary resistance to ICIs therapy.

In addition to the immune cells that influence the therapeutic efficiency of ICIs, there are also some inhibitory molecules and cytokines in the TME. In epithelial cells, transforming growth factor Β (TGF-Β) has a tumor suppressor effect. In other contexts, however, TGF-Β has the effect of promoting tumor invasion and metastasis. Its tumor-promoting effect is not only limited to tumor cells but also affects the TME (43). Principe et al. reported that TGF-Β reduces T cell infiltration and impairs granzyme B-mediated tumor destruction (44). Moreover, TGF-Β plays an immunosuppressive role independently of Foxp3^+^Tregs (45). In the hypoxic tumor microenvironment, CD39 and CD73 stimulate the conversion of ATP to adenosine, which weakens antitumor immunity (46, 47). In a preclinical study, researchers observed that treatment targeting CD73 alleviated the suppression of lymphocytes by adenosine and inhibited tumor growth in mice *in vivo*. Therapy targeted against the adenosinergic pathway combined with anti-PD-1 therapy is more effective than monotherapy (48). In addition, vascular epithelial growth factor (VEGF) (49) and indoleamine 2,3-dioxygenase (IDO) (50) can also induce the function of Tregs, leading to immunosuppression. CD8^+^T cells in the TME can kill tumor cells by secreting IFN-γ, which exerts its effects through the downstream JAK/STAT pathway (51). Furthermore, IFN-γ can inhibit tumor progression by increasing tumor apoptosis and recruiting other immune cells to limit tumor cell proliferation (52). However, IFN-γ is also a double-edged sword in the anti-tumor immune response, since it promotes the upregulation of PD-L1 in the TME and consequently decreases the activity of CD8^+^T cells (53). Mutations in IFN-γ pathway-related proteins (such as JAK1/2 and STAT1) can also result in resistance of tumor cells to anti-PD-1/PD-L1 therapy (54, 55).

The expression of other inhibitory receptors can also contribute to T cell depletion, resulting in anti-PD-1/PD-L1 therapy resistance. These include CTLA4 (56), lymphocyte-activation gene 3 (LAG3) (57), T-cell Ig and mucin domain-3 protein (TIM3) (58), as well as B and T lymphocyte attenuator (BTLA) (59). These compensatory inhibitory signals are elevated throughout therapy, which makes the anti-PD-1/PD-L1 treatment unable to achieve the best therapeutic effect, resulting in acquired drug resistance. Therefore, anti-PD-1/PD-L1 therapy can only overcome part of the inhibitory signals in the TME, and there may be more inhibitory axes that play a role in suppressing T cell function in the TME.

#### 2.2.3 Abnormal expression of PD-L1

Low expression of PD-L1 leads to the development of primary resistance, thereby reducing the efficacy of treatment. In a clinical trial of NSCLC, the expression of PD-L1 in more than 50% of tumor cells was related to higher pembrolizumab effectiveness (60). Conversely, an increase in PD-L1 expression may also diminish the effectiveness of anti-PD-1/PD-L1 therapy. Theodoraki et al. demonstrated that patients with high levels of PD-L1^+^exosomes in their plasma had a worse prognosis (61). IFN-γ stimulates the expression of PD-L1 in exosomes, and these PD-L1^+^exosomes impair the function of CD8^+^T cells, promote tumor progression, and trigger immune escape (62). Tumor-infiltrating T cells release IFN-γ, which increases the expression of PD-L1 on the surface of tumor cells or other immune cells infiltrating the tumor. Overexpressed PD-L1 binds to PD-1 on the surface of T cells, hence restricting the effector function of T cells (63). At the same time, several lncRNAs and circular RNAs were found to boost the expression of PD-L1 in tumor cells and promote tumor resistance to anti-PD-1 therapy (64–67). Therefore, the increase or decrease of PD-L1 expression in the tumor microenvironment may affect the efficacy of anti-PD-1/PD-L1 therapy, which can be explained by different immune evasion mechanisms. Furthermore, the expression of PD-L1 in tumor tissue may be heterogeneous, and the results of a single biopsy cannot represent the overall response of the tumor to anti-PD-1/PD-L1 therapy. Therefore, future research should further explore the connection between PD-L1 expression and anti-PD-1 effectiveness.

### 2.3 Strategies for overcoming drug resistance

As a result of the advancement of cancer research, different therapeutic techniques have been proposed for various causes of drug resistance in tumors. Resistance to anti-PD-1/PD-L1 therapy may emerge at various stages of cancer treatment. Therefore, it is essential to combine anti-PD-1/PD-L1 therapy with other therapeutic techniques. Currently, therapeutic strategies for overcoming drug resistance mainly focus on increasing the recognition of tumor cells by the immune system (68–71), enhancing T cell infiltration in tumor tissue (72, 73), reversing the exhausted state of T cells (74), and improving the immunosuppressive tumor microenvironment (75, 76). Regorafenib has been proven in an increasing number of studies to improve the TME and boost the anti-tumor immune response, hence reducing anti-PD-1/PD-L1 treatment resistance.

## 3 Immunomodulatory effect of regorafenib

The TME comprises multiple cell types, including immune cells, fibroblasts, neurons, adipocytes, and endothelial cells, as well as extracellular components, including the extracellular matrix, cytokines, growth factors, chemokines, and extracellular vesicles (77, 78). The TME is strongly immunosuppressive, which is why most antitumor therapies targeting tumor cells have limited therapeutic effects. Multiple immunosuppressive cells in the TME facilitate the evasion of anticancer immune responses by tumor cells. TAMs, which are major immunosuppressive cells, can not only promote tumor invasion and metastasis but also promote tumor angiogenesis (79). MDSCs, as a heterogeneous population of immature myeloid cells, are also reported to be involved in resistance to anti-VEGF treatment (80). Tregs in the TME decrease anti-tumor immunity *via* numerous mechanisms, including directly inhibiting immune cells, interacting with antigen-presenting cells and fibroblasts, secreting cytokines, etc. (81). Moreover, tumor-associated neutrophils (TANs), cancer-associated fibroblasts (CAFs), and tumor-associated endothelial cells (TECs) in the TME all predict poor prognosis in a variety of cancers (82–84). The extracellular matrix also plays an important role in tumor resistance and the promotion of tumor invasion and metastasis. The TME influences the acquisition and maintenance of tumor hallmarks to varying degrees. This dependency of tumor cells on the TME presents a potential for anticancer therapy. Targeting the TME provides additional advantages over traditional therapeutic strategies (78). Transforming a tumor’s immunosuppressive microenvironment into an immunostimulatory microenvironment enables the immune system to eliminate malignancies (85). Stimulation of the antitumor immune response by regorafenib can augment the efficacy of anti-PD-1/PD-L1 treatment and exert a more potent antitumor effect.

Regorafenib targets multiple tyrosine kinases, including vascular endothelial growth factor receptors (VEGFR)1-3, platelet-derived growth factor receptor (PDGFR), fibroblast growth factor receptors (FGFR)1 and 2, tyrosine kinase with immunoglobulin-like and epidermal growth factor-like domains 2 (TIE2), the oncogenic kinases RET and KIT, rapidly accelerated fibrosarcoma (RAF), as well as colony-stimulating factor-1 receptor (CSF1R) (86). These targets have significant roles in tumor angiogenesis, promoting tumor growth and metastasis. Regorafenib alleviates tumor resistance to anti-PD-1/PD-L1 treatment in several aspects through diverse targets, regulates the TME, and reduces tumor progression.

### 3.1 Effects on natural killer (NK) cells

NK cells are the primary effector cells in innate anticancer immunity and have the ability to autonomously kill target cells. The majority of treatment approaches targeting the tumor microenvironment concentrate on T cell immunity while ignoring the role of NK cells (87). NKG2D, a key receptor on NK cells, can recognize different ligands on tumor cells, including MHC class I polypeptide-related sequence A (MICA), to exert an antitumor effect. The combination of NKG2D and MICA on tumor cells can enhance the tumor-killing activity and cytokine production of NK cells (88). However, several studies have observed that human tumor cells release soluble MICA by proteolytic shedding and that these sMICA molecules result in the downregulation of NKG2D and impede NKG2D-mediated tumor immune surveillance (89–91). ADAM10 and ADAM9 are sheddases that release MICA from the cell membrane of tumor cells. Regorafenib can suppress the transcriptional expression of ADAM10 and ADAM9 and diminish the protein levels of sMICA, thereby improving the function of NK cells in antitumor immunity (92, 93).

In addition, it has been observed that the overexpression of CD24 correlates with the invasiveness and metastasis capacity of malignancies (94), while the activation of STAT3 is related to the expression of CD24 (95, 96), and inhibition of p-STAT3 can limit the expression of CD24 (97). Interestingly, in experiments with liver cancer cell lines and mouse subcutaneous tumors, regorafenib inhibited p-STAT3 by boosting the activity of SHP-1, thereby inducing the apoptosis of tumor cells (98). Siglec-10 is an immunosuppressive receptor expressed on monocytes, eosinophils, and NK cells (99–101). The combination of CD24 and Siglec-10 impairs NK cell function and decreases survival in patients with HCC (102). Therefore, regorafenib may influence the expression of CD24 by modulating the expression of p-STAT3, further affecting the function of NK cells.

### 3.2 Effects on TAMs

Regorafenib can alleviate the inhibitory tumor microenvironment by suppressing TAMs in the TME through specific mechanisms. There are TIE2-expressing macrophages (TEMs) in tumors, which can accelerate tumor angiogenesis and tumor progression (103). Targeting the ANG2/TIE2 axis has been shown to reduce angiogenesis and tumor growth in preclinical studies (104). Regorafenib can inhibit the infiltration of TAMs by inhibiting the TIE2 pathway (105), while inducing sustained M1 polarization and reversing M2 polarization (106–110). Bai et al. demonstrated that regorafenib not only inhibited the activation of M2 macrophages but also caused M2 inactivation by reducing the production of tumor-suppressive cytokines (111). Moreover, the p38MAPK pathway plays a vital role in tumor development (112). In *in vitro* experiments, regorafenib promoted antitumor immunity *via* the p38MAPK/Creb1/Klf4 pathway. In macrophages, regorafenib reduced the phosphorylation of p38MAPK and Creb1, while increasing the expression of Klf4 and M1-related genes (113). In a model of colorectal cancer in mice, regorafenib reduced the abundance of TAMs in a dose-dependent manner through inhibition of CSF1R (114).

Doleschel et al. demonstrated that when treatment was discontinued after 10 days of continuous treatment, the regorafenib combined with anti-PD-1 treatment group still maintained tumor suppression, and the tumor volume increased only slightly until the end of the observation period (108). After withdrawal of treatment, macrophages increased in the group receiving combination treatment, and the combination therapy led to a polarization of tumor-associated macrophages towards the M1 phenotype. These results indicate that the addition of anti-PD-1 therapy to regorafenib therapy can improve the microenvironment of tumors and prevent rapid tumor regeneration (108).

### 3.3 Effects on CD8^+^T cells and Tregs

In addition to activating NK cells and affecting the polarization of tumor-associated macrophages, regorafenib also induces CD8^+^T cell activation and suppresses Tregs. One study showed that regorafenib increased the expression of CXCL10 in HCC cells by inhibiting the activity of STAT3. As a ligand of CXCR3, CXCL10 can mediate the infiltration of T cells into tumor tissues. Furthermore, regorafenib combined with anti-PD-1 treatment increased the number of CXCR3^+^CD8^+^T cells in tissues, enhancing the effector role of T cells (115). Although both regorafenib and DC-101 (VEGFR2 receptor inhibitor) inhibit tumor angiogenesis, regorafenib induces tumor infiltration by CD4^+^ and IFN-γ^+^CD8^+^T cells, which is associated with better antitumor efficacy (113). In addition to HCC models, regorafenib combined with ICIs also produces significant antitumor effects in other cancers, such as melanoma (116), CRC (108), and oral squamous cell carcinoma (OSCC) (109). In an orthotopic OSCC model, IFN-γ^+^IL-2^+^CD8^+^T cell infiltration was considerably higher in the regorafenib combined anti-PD-L1 therapy group than in the monotherapy group or the control group. From improving the immunosuppression status to enhancing immune activation, regorafenib further enhanced the therapeutic effect of ICIs in OSCC (109). In addition, through their immunosuppressive effects, CD4^+^ CD25^+^ Foxp3^+^ Tregs play a crucial role in the development and prognosis of HCC (117). The amount of Tregs in the peripheral blood of HCC patients is higher than that of the healthy control group (118). In addition, an increase in Tregs in HCC tumors predicted a poorer outcome (119–121). By secreting cytokines like interleukin-10 (IL-10) and TGF-Β, Tregs in the TME exert immunosuppressive effects (122, 123). Importantly, Tregs express inhibitory receptors like CTLA4, PD-1, and others, making them potential direct targets for improving the TME (117). Regorafenib has been reported to significantly reduce Treg infiltration in the TME (108). Chiang et al. reported that regorafenib combined with anti-PD-L1 treatment decreased the accumulation of Tregs and MDSCs (109). Additionally, suppressive immune cells such as TAMs, MDSCs, and CAFs in the TME promote the accumulation of Tregs (124–126). Therefore, regorafenib may inhibit Tregs by acting on these suppressive immune cells.

### 3.4 Effects on CAFs

CAFs also play a crucial role in inhibiting antitumor immunity. CAFs recruit inhibitory immune cells to promote tumor growth by secreting inhibitory cytokines. For example, CAFs recruit and activate Tregs by secreting regulators such as CXCL12 and CD73, thereby suppressing the function of effector T cells and promoting the formation of an immunosuppressive microenvironment (125). In addition, CAFs recruit MDSCs and TAMs through the secretion of TGF-Β and complement C5a, thereby boosting their potential to suppress effector T cells and promote tumor growth (127–130). Consequently, suppressing CAFs in the TME is also a method for improving immunotherapy.

Zhang et al. reported that regorafenib suppressed the proliferation and promoted the apoptosis of CAFs by acting on the AKT/Bcl-2/Bax pathway *in vitro*. Moreover, *in vivo* experiments revealed that regorafenib reduced TAMs infiltration in tumors and inhibited tumor growth by targeting CAFs (131). In a nude mouse model with co-implanted colon cancer cells and bone marrow mesenchymal stem cells, regorafenib had a more potent antitumor effect than in colon tumor-bearing mice. In both tumor models, pERK1/2 expression was reduced by regorafenib. Studies have shown that regorafenib inhibits not only the interaction between tumor cells and CAFs, but also reduces tumor angiogenesis and lymphangiogenesis, thus decreasing the invasion and metastasis of colon cancer (132).

### 3.5 Effects on PD-L1 and MCH-I expression

A study evaluating the effects of 161 different kinase inhibitors on melanoma cell lines found that regorafenib is the only drug that reduces the expression of PD-L1 in tumor cells without decreasing the amount of MHC-I on the surface of the cell (116). Regorafenib indirectly inhibits JAK1/2-STAT1 signaling by inhibiting the oncogenic tyrosine kinase RET. Interestingly, RET/Src signaling showed no influence on MHC-I levels, while suppression of RET/Src signaling by regorafenib reduced IFN-γ-induced expression of PD-L1 and IDO1 on tumor cells. The expression of PD-L1 facilitates tumor cell evasion of T-cell effector functions, but regorafenib alleviates immune evasion by inhibiting IFN-γ-induced PD-L1 expression (116). However, in another preclinical investigation, the researchers discovered that in the presence of IFN-γ, regorafenib could upregulate the expression of MCH-I on HCC cells by acting on the IFN-γ/STAT1 signaling pathway (133). At the same time, regorafenib enhanced the expression of MCH-I transcriptional regulators, such as IRF1 and NLRC5. In terms of up-regulating MHC-I, regorafenib was more effective than lenvatinib, sorafenib, and cabozantinib. This may be due to the fact that regorafenib has a more potent inhibitory impact on the MEK/ERK pathway (133). Increasing numbers of studies indicate that regorafenib can impact the expression of PD-L1 and MCH-I. Nevertheless, further efforts are needed to understand the underlying mechanisms.

### 3.6 Inhibition of angiogenesis

The increase of angiogenic factors such as VEGF and ANG2 in the tumor microenvironment might result in aberrant tumor angiogenesis and structure, as well as facilitate tumor evasion of immunotherapy (134). When tumor blood vessels function abnormally, immune cells in tumor tissue are inhibited by various factors, blocking their normal antitumor effect. Firstly, the hypoxic tumor microenvironment facilitates the infiltration of MDSCs and TAMs and induces the polarization of TAMs toward the M2 type (135). Additionally, the elevated expression of CC-chemokine ligand 28 (CCL28) in a hypoxic environment indirectly results in the accumulation of Tregs in the tumor microenvironment (136). Secondly, the hypoxic environment promotes the expression of CTLA4 and TIM3 on Tregs and stimulates the expression of PD-L1 on tumor cells (137, 138). Therefore, anti-tumor angiogenesis alleviates hypoxia by improving tissue perfusion, enhances the infiltration of the tumor by immune cells, and alleviates the immunosuppressive environment, which is crucial for antitumor therapy.

**Figure 1:**
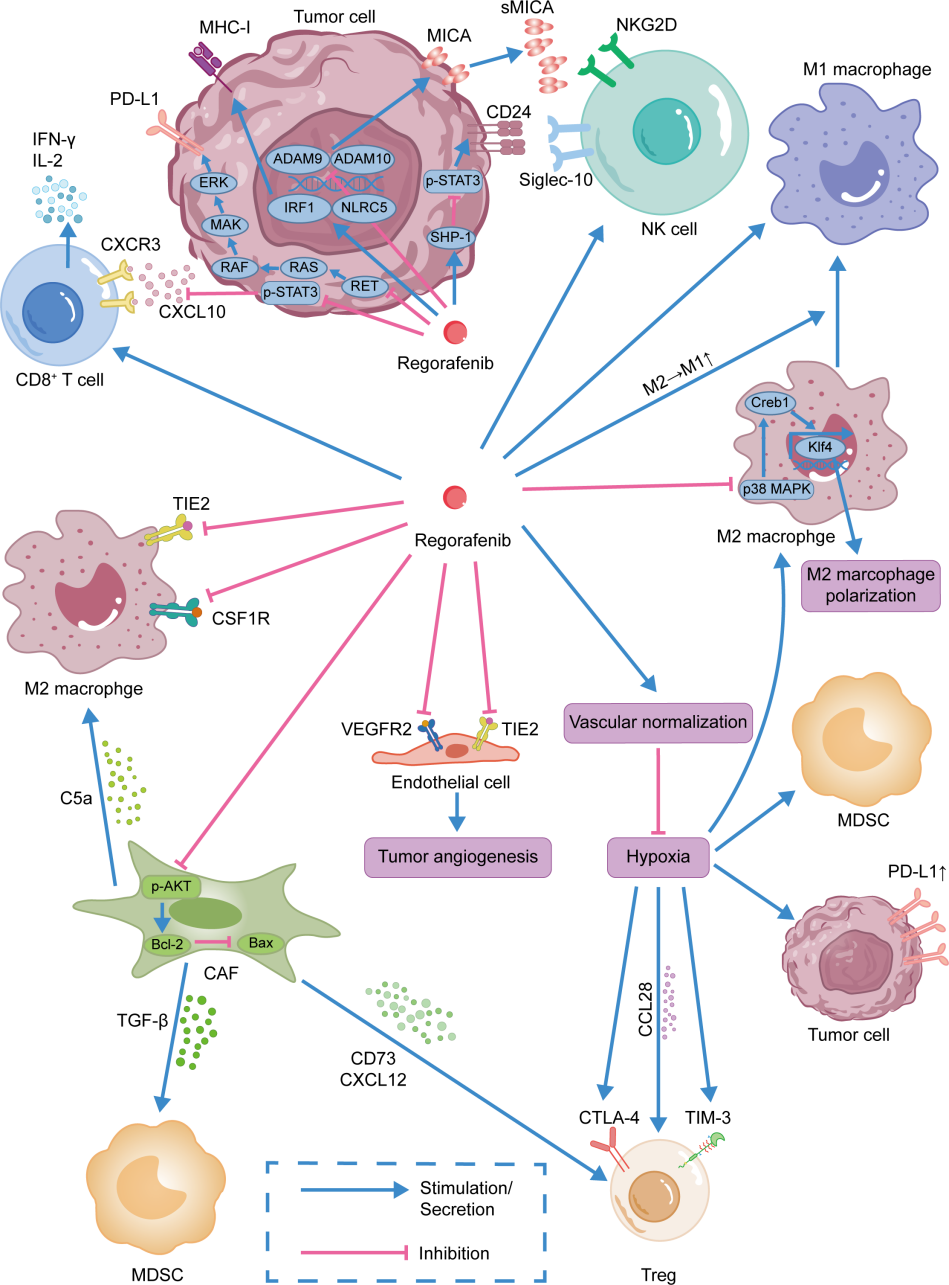
Immunomodulatory effect of regorafenib.Regorafenib was shown to promote the infiltration of CD8^+^ T cells into tumor tissues and activate NK cells. By normalizing tumor blood vessels, regorafenib improves the immunosuppressive tumor microenvironment. Moreover, regorafenib was also shown to inhibit MDSCs, CAFs, and Tregs and promote the repolarization of TAMs from M2-like to M1-like phenotype.

Regorafenib has been shown to be effective in inhibiting angiogenesis. For example, regorafenib reduced tumor angiogenesis in a mouse orthotopic colon cancer model by targeting the VEGFR2 and TIE2 receptors. The ratios of VEGFR2^+^/CD31 and TIE2^+^/CD31 in regorafenib-treated tumors were considerably lower than in the control group (105). Regorafenib also inhibits tumor angiogenesis, which slows the growth of diverse malignancies, including colon cancer (132), breast cancer (139), gastric cancer(GC) (140), and HCC (141). Notably, the original idea was that antiangiogenic drugs could limit oxygen and nutrient delivery to tumors (142). However, excessive inhibition of tumor blood vessels might result in tumor invasion and metastasis by inducing transformation of the tumor into a hypoxia-tolerant phenotype (143). Thus, Jain et al. proposed the notion of normalization of tumor vasculature. Normalization of blood vessels can alleviate hypoxia and enhance the delivery of antitumor medications. Therefore, the application duration and dose of antiangiogenic medicines merit more research (142). In the above-mentioned study of regorafenib combined with anti-PD-1 therapy, researchers discovered that in an orthotopic mouse model of HCC, an intermediate dose (10 mg/kg) of regorafenib combined with anti-PD-1 therapy considerably decreased the region of hypoxia in comparison to other doses of regorafenib. Through the use of 5, 10, and 20 mg/kg of regorafenib in combination with anti-PD-1 therapy in the treatment of the orthotopic mouse model, it was discovered that an intermediate dose of regorafenib can promote the normalization of intratumoral blood vessels, thereby increasing the delivery of therapeutic drugs (115). More research is required to determine the optimal dose and treatment duration of these anti-angiogenic medicines for the treatment of malignancies. (**Figure 1**)

## 4 Clinical trials of combined therapy

Regorafenib in combination with anti-PD-1/PD-L1 therapy has been utilized in a growing number of clinical trials. (**Table 1**) The clinical outcomes of the studies in **Table 1** vary to some extent, possibly because the studies are still in the early stages and there are differences in the subjects selected for different studies. Therefore, more clinical trials are needed to verify the efficacy of the combination therapy.

**Table 1:** Clinical trials of combined therapy.

Therapeutic drug	Phase	n	Study population	ORR	Median OS (95%CI), months	Median PFS (95%CI), months
regorafenib plus nivolumab (9)	I/Ib	52	pMMR metastatic CRC	10%	11.1 (9.7-NE)	4.3 (2.3-7.9)
regorafenib plus nivolumab (144)	Ib	50	advanced GC and CRC	GC:44% CRC:36%	GC:12.3 (5.3-NR) CRC : NR (9.8-NR)	GC:5.6 (2.7-10.4) CRC:7.9 (2.9-NR)
regorafenib plus nivolumab (157)	II	70	pMMR/MSS metastatic CRC	7.1%	12.1	1.9
regorafenib and pembrolizumab (158)	II	73	pMMR/MSS metastatic CRC	–	10.9 (5.3-NR)	2.0 (1.8-3.5)
regorafenib plus toripalimab (159)	Ib/II	42	metastatic CRC	15.2%	15.5 (10.3-NR)	2.1 (2.0–4.3)
regorafenib plus avelumab (149)	II	34	advanced BTC	–	11.9 (6.2-NA)	2.5 (1.9-5.5)
regorafenib plus avelumab (160)	II	48	microsatellite Stable CRC	–	10.8 (5.9-NA)	3.6 (1.8-5.4)

CRC, colorectal cancer; GC, gastric cancer; BTC, biliary tract cancer; MSS, microsatellite stable; pMMR, mismatch repair proficient; ORR, objective response rate; OS, overall survival; PFS, progression-free survival; NE, not estimable; NR, not reached; NA, not attained.

In REGONIVO trial in CRC patients, the median PFS was 7.9 months (95% confidence interval [CI], 2.9 months-NR), and the objective response rate (ORR) was 36% (95%CI, 18.0%-57.5%) (144). Compared with regorafenib (ORR: 3%) in the INTEGRATE trial (150) or Nivolumab (ORR: 11.9%) in the ATTRACTION-2 trial (151), combination therapy provided greater benefits than monotherapy. In addition, among GC patients, median PFS and median OS were 6.5 months (95% CI, 2.7 months-10.4 months) and 12.3 months (95% CI, 5.3 months-NR), respectively. With regorafenib and nivolumab, three of seven patients with refractory GC who had previously undergone anti-PD-1 monotherapy achieved a response (144). This study demonstrated that regorafenib may overcome anti-PD-1 treatment resistance, and the combination of regorafenib with anti-PD-1 therapy has promising therapeutic potential. In addition, the combination therapy also showed good efficacy and tolerable safety in the treatment of mismatch repair proficient (pMMR) CRC (9). In this phase IB study, 52 patients were recruited to receive the regorafenib and nivolumab combination. The median progression-free survival (PFS) and overall survival (OS) for the 40 evaluable patients were 4.3 and 11.1 months, respectively. Overall, 10 percent of the 40 evaluable patients showed a partial response, and 53 percent attained stable disease. In this clinical experiment, the researchers found that the expression of Treg in tumor tissue was negatively correlated with the FPS of patients (9). The results of the REGONIVO trial mentioned above are better than this clinical trial. This discrepancy may be due to differences in the patients included in the two trials. Therefore, researchers still need to explore more sensitive biomarkers for predicting the efficacy of combination therapy.

Patients with biliary tract cancer (BTC) often have a dismal prognosis, with few therapeutic options available after the failure of first-line therapy, with the limited effectiveness of ICI therapy alone (152, 153). However, research suggests that regorafenib may improve the efficacy of ICIs in BTC patients. In a phase II multicenter, single-arm, open-label trial of regorafenib plus avelumab (anti-PD-L1 antibody), the combination treatment demonstrated promising effects against cholangiocarcinoma. Four (13.8%) of the twenty-nine evaluable patients achieved partial responses, eleven (37.0%) achieved stable disease, and fourteen (48.3%) had progressive disease. The median PFS was 2.5 months (95%CI, 1.9 months-5.5months) and overall survival was 11.9 months (95%CI, 6.2months-NA). In this trial, the researchers also found that patients with higher expression of PD-L1 and IDO1 had better treatment outcomes (148). These results indicate which patients with cholangiocarcinoma might benefit most from regorafenib in combination with ICIs.

In addition to CRC, GC, and BTC, the combination of Regorafenib and anti-PD-1/PD-L1 therapy has also shown promising therapeutic effects in HCC. In a multicenter retrospective study of advanced HCC, researchers evaluated the efficacy of regorafenib plus sintilimab versus regorafenib alone. Combination therapy has longer FPS, higher ORR, and better OS than monotherapy. The median PFS for the combination therapy was 5.6 months (95% CI, 4.2 months–7.0 months) and the median OS was 13.4 months (95% CI, 9.2 months–17.5 months). The ORR of combination therapy was 24.1%, compared with 9.1% for monotherapy (154). In another phase Ib clinical trial of regorafenib plus pembrolizumab for first-line treatment of advanced HCC, the disease control rate (DCR) was 88% in the regorafenib 120 mg group and 91% in the regorafenib 80 mg group (RECIST v1.1) (155).

In conclusion, the combination of regorafenib with anti-PD-1/PD-L1 treatment may have a more potent impact than monotherapy in modifying the tumor microenvironment and reducing angiogenesis. However, further research is necessary to enable an informed choice of the most effective therapy based on the unique features of each patient. Currently, there are many ongoing clinical trials of the combination therapy for the treatment of cancer, including the evaluation of combinations with nivolumab, sintilimab, and toripalimab. (**Table 2**)

**Table 2:** Ongoing trials of regorafenib combined with anti-PD-1/PD-L1 therapy.

anti-PD-1/PD-L1 therapy	Cancer types	Clinical trials identifier	Clinical phase	Status	Primary outcome measures
Camrelizumab/Toripalimab/Pembrolizumab	HCC	NCT05048017	II	Recruiting	PFS
Nivolumab	MMR Refractory CRC	NCT03712943	I	Active, not recruiting	MTD
Nivolumab	HCC	NCT04170556	I/II	Recruiting	Safety
Durvalumab	high-risk HCC	NCT05194293	II	Not yet recruiting	ORR
Nivolumab	unresectable HCC	NCT04310709	II	Recruiting	Response rate
Pembrolizumab	advanced or metastatic CRC	NCT03657641	I/II	Recruiting	PFS, OS, DLTs
Nivolumab/Carelizumab/Sintilimab/Toripalimab	CRC	NCT04110093	I/II	Recruiting	ORR, PFS
Carelizumab	HCC	NCT04806243	II	Recruiting	OS
Nivolumab	Osteosarcoma	NCT04803877	II	Recruiting	PFS
Durvalumab	advanced BTC	NCT04781192	I/II	Recruiting	PFS, Safety
Pembrolizumab	advanced metastatic HCC	NCT04696055	II	Active, not recruiting	ORR
Nivolumab	solid tumors	NCT04704154	II	Recruiting	ORR
Pembrolizumab	HCC	NCT03347292	I	Active, not recruiting	Safety

HCC, hepatocellular carcinoma; CRC, colorectal cancer; BTC, biliary tract cancer; PFS, progression free survival; MTD, maximum tolerated dose; ORR, objective response rate; OS, overall survival; DLTs, dose limiting toxicity; MMR, mismatch repair.

## 5 Discussion and future perspectives

**Figure 2:**
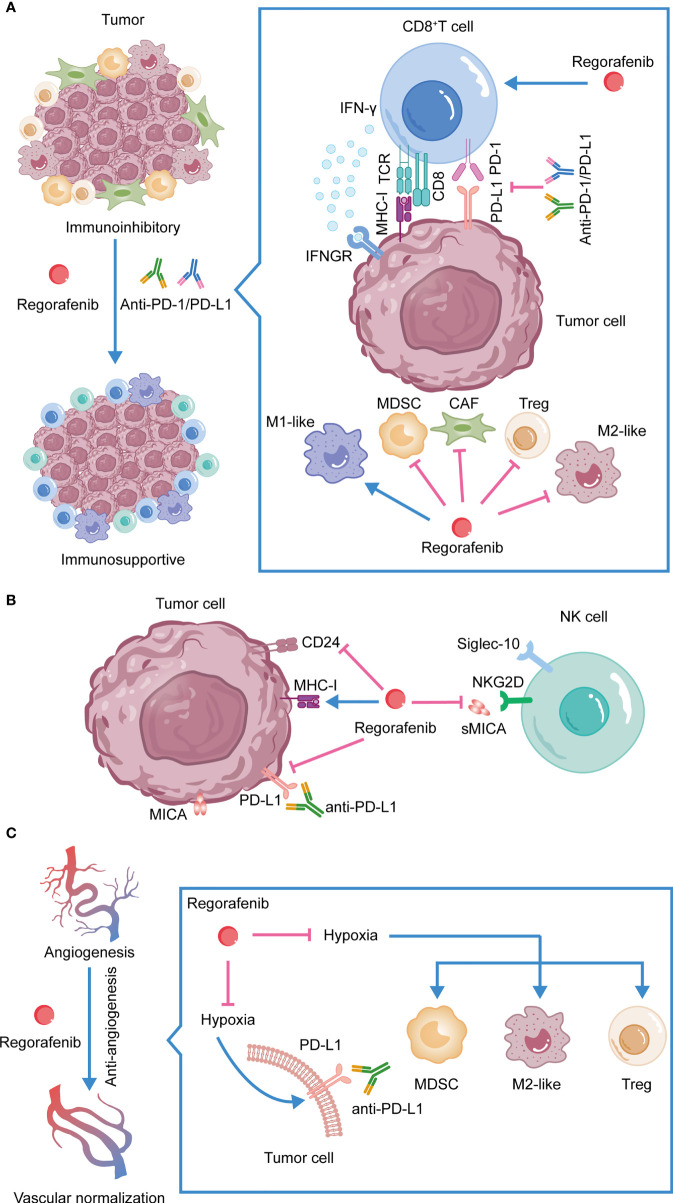
The synergistic antitumor efficacies and mechanisms of anti-PD-1/PD-L1 in combination with regorafenib. **(A) **Combination therapy reshapes the TME. **(B)** Regorafenib activates NK cells, inhibits the expression of PD-L1 on tumor cells and promotes the expression of MHC-I. **(C)** Regorafenib promotes vessel normalization, enhances drug delivery, alleviates hypoxia-induced PD-L1, reduces hypoxia-induced infiltration of suppressive immune cells, and increases the efficacy of anti-PD-1/PD-L1 therapy.

Anti-PD-1/PD-L1 therapy has shown significant effects in the treatment of several malignancies. However, the emergence of primary or acquired tumor resistance indicates that not all patients receive therapeutic benefits. As a tyrosine kinase inhibitor, regorafenib has an effect on the TME in addition to its involvement in inhibiting tumor cell invasion and metastasis, as well as promoting tumor cell apoptosis. Firstly, Regorafenib enhances antitumor immunity by leading to a polarization of tumor-associated macrophages towards the M1 phenotype and by increasing the proliferation and activation of CD8^+^ T cells (105, 113). Secondly, regorafenib decreases the accumulation of Tregs and MDSCs, thereby alleviating the suppressive TME (109). In the TME, TAMs, MDSCs, and CAFs all promote the accumulation of Tregs (124–126). Consequently, regorafenib may inhibit Tregs by acting on these immunosuppressive cells. By inhibiting CAFs’ proliferation and promoting apoptosis, regorafenib also inhibits tumor cell invasion and metastasis (131). Thirdly, regorafenib affects the function of NK cells by inhibiting the expression of sMICA and CD24 on tumor cells (92, 93, 97, 98). Moreover, regorafenib increases MHC-I expression on tumor cells, thus enhancing the immunogenicity of the tumor. Regorafenib also reduces immune evasion by suppressing the expression of PD-L1 on tumor cells (116, 133). Fourthly, by acting on receptors such as VEGFR2 and TIE2, regorafenib promotes intratumoral blood vessel normalization, enhances drug delivery, reduces hypoxia-induced infiltration of suppressive immune cells, and prevents hypoxia-induced PD-L1 upregulation (115, 138). The immunomodulatory actions of regorafenib reduce resistance to anti-PD-1/PD-L1 treatment in multiple ways, which illustrates the therapeutic significance of their combination. (**Figure 2**)

Regorafenib combined with anti-PD-1/PD-L1 therapy is one of the trendiest issues in tumor therapy research in recent years. A patient with lung metastases from HCC who underwent a combination of regorafenib and sintilimab had a complete response of tumor lesions in the liver and lungs, which we previously reported (156). Zhang et al. also reported that a patient with small bowel adenocarcinoma received nivolumab plus regorafenib after chemotherapy and targeted therapy failed, and the symptoms were significantly relieved, and the disease was stable for 15 months (157). In addition, Yong et al. reported that a metastatic rectal adenocarcinoma patient also experienced a complete response after treatment with regorafenib and sintilimab (158). However, the combination therapy also confronts some obstacles. Future research should investigate how to obtain precise treatment, identify specific and extremely sensitive biomarkers, and choose the most suited individuals for combination therapy. A recent study has revealed possible biomarkers for predicting OS in regorafenib-treated patients by analyzing proteins and microRNAs in baseline plasma samples from patients with different responses. Some proteins and microRNAs, such as ANG-1 and MIR122, can be utilized as potential markers to predict the OS of cancer patients (159). Another study demonstrated that a signature composed of five biomarkers (HIF1A, CDKN1A, miR-3607-3p, miR-301a-3p, and miR-93-5p) in glioblastoma patients with regorafenib treatment can be used to identify patients with a survival advantage (160). These biomarkers might effectively predict the response of cancer patients to regorafenib. However, due to the heterogeneity of tumors, the expression of certain proteins may not accurately predict the action of medications on the entire tumor, which will increase the difficulty of finding suitable biomarkers. In any event, the discovery of reliable biomarkers for predicting the efficacy of combination therapy remains the path of future research, which will allow us to identify the most suitable patients for combination therapy.

## 6 Conclusions

This review outlined the underlying mechanism of resistance to PD-1/PD-L1 blockade and the immunomodulatory effect of regorafenib, providing a theoretical basis for regorafenib combined with anti-PD-1/PD-L1 therapy. Regorafenib was shown to interact with NK cells, TAMs, cytotoxic T cells, Tregs, and CAFs in the TME. Additionally, regorafenib affects PD-L1 and MHC-I on tumor cells and normalizes abnormal blood vessels. From traditional chemotherapy to molecular targeted therapy and combination immunotherapy, the area of anticancer therapy has witnessed several significant developments. There are ever more therapies available to alleviate the patient’s symptoms and improve their prognosis. Regorafenib in combination with anti-PD-1/PD-L1 therapy has shown excellent antitumor efficacy and tolerable safety in the treatment of a variety of malignancies. The study of the synergetic mechanism of combination therapy and the identification of possible biomarkers are worthwhile future research paths that will help us identify the most suitable individual patients for more precise treatment.

## Author contributions

All authors contributed to the article and approved the submitted version of the manuscript.

## Funding

This project was supported by Hubei provincial special grants for scientific and technical innovation (No.2021BCA115) and the National Natural Science Foundation of China (No. 81902839).

## Conflict of interest

The authors declare that the research was conducted in the absence of any commercial or financial relationships that could be construed as a potential conflict of interest.

## Publisher’s note

All claims expressed in this article are solely those of the authors and do not necessarily represent those of their affiliated organizations, or those of the publisher, the editors and the reviewers. Any product that may be evaluated in this article, or claim that may be made by its manufacturer, is not guaranteed or endorsed by the publisher.
